# The Effect of Heat Treatment on the Gene Expression and Cryosurvival of In Vitro-Produced Bovine Embryos

**DOI:** 10.3390/ani16152289

**Published:** 2026-07-23

**Authors:** Katalin Nagy, Nikolett Tokodyné Szabadi, Elen Gócza, Bence Lázár, András Ecker, Arnold Tóth, Lilla Sándorová, Viktor Stéger, Alexandra Tokár, Delgado Gustavo, Zoltán Zomborszky, Szilárd Bodó

**Affiliations:** 1 Department of Precision Livestock Farming and Animal Biotechnics, Institute of Animal Sciences, Hungarian University of Agriculture and Life Sciences, H-7400 Kaposvár, Hungary; nagy.katalin.eti@uni-mate.hu (K.N.); tokar.alexandra@uni-mate.hu (A.T.); bodo.szilard@uni-mate.hu (S.B.); 2 Doctoral School of Agricultural and Food Sciences, Hungarian University of Agriculture and Life Sciences, H-7400 Kaposvár, Hungary; toth.arnold.2@phd.uni-mate.hu (A.T.); sandorova.lilla@uni-mate.hu (L.S.); delgado.quinonez.gustavo.harrison@phd.uni-mate.hu (D.G.); 3 Department of Animal Biotechnology, Institute of Genetics and Biotechnology, Hungarian University of Agriculture and Life Sciences, H-2100 Gödöllő, Hungary; tokodyne.szabadi.nikolett@uni-mate.hu (N.T.S.); lazar.bence@uni-mate.hu (B.L.); 4 Institute for Farm Animal Gene Conservation, National Centre for Biodiversity and Gene Conservation, 200 Isaszegi Street, H-2100 Gödöllő, Hungary; 5 Institute of Aquaculture and Environmental Safety, Hungarian University of Agriculture and Life Sciences, H-2100 Gödöllő, Hungary; ecker.andras@uni-mate.hu; 6 Department of Genetics and Genomics, Institute of Genetics and Biotechnology, Hungarian University of Agriculture and Life Sciences, H-2100 Gödöllő, Hungary; steger.viktor@uni-mate.hu; 7 9-Life Veterinary Clinic, H-7400 Kaposvár, Hungary; zomborszky.zoltan@gmail.com

**Keywords:** bovine, embryo, heat treatment, cryopreservation, miR-378, *HSP90AA1*, *HSPB11*

## Abstract

Heat stress negatively affects bovine fertility and embryonic development, while cryopreservation imposes additional cellular stress that may impair embryo survival. The present study investigated whether controlled heat treatment applied during the early morula stage could influence subsequent blastocyst development, molecular stress responses, and post-thaw survival of bovine embryos. In vitro-produced bovine embryos were exposed to controlled thermal treatment involving gradual elevation in temperature from 38.8 °C to 40.0 °C followed by subsequent cooling. Blastocyst formation, post-thaw survival after slow freezing, and the expression of stress-associated genes and microRNAs were evaluated. Heat treatment did not significantly affect blastocyst formation rates or post-thaw embryo survival compared to the control groups. However, significant alterations in the expression of stress-associated markers were observed. The expression of HSP90AA1 and HSPB11 was significantly reduced following heat treatment, while cryopreservation induced increased expression of these markers compared to embryos exposed only to heat treatment. Significant alterations were also detected in the expression of several stress-associated microRNAs. These findings suggest that controlled heat exposure during the morula stage may induce molecular changes associated with the embryonic response to heat stress without compromising developmental competence or cryosurvival. The results contribute to a better understanding of the molecular mechanisms underlying embryonic responses to thermal and cryogenic stress and may support future optimization of embryo production and cryopreservation strategies.

## Simple Summary

High environmental temperatures can negatively affect fertility and embryo survival in cattle, creating important challenges for animal production worldwide. This study investigated whether carefully controlled and mild heat exposure applied to bovine embryos during early development could help the embryos better tolerate later stress conditions, such as freezing and thawing procedures commonly used in reproductive technologies. The results showed that the applied heat treatment did not significantly reduce embryo development or survival after freezing. In addition, several stress-related genes and small regulatory molecules changed their activity patterns after treatment, suggesting that embryos may activate protective mechanisms rather than showing signs of severe damage. The observed alterations in gene and miRNA expression provide new insights into the molecular responses associated with heat exposure and cryopreservation. These findings may contribute to a better understanding of the mechanisms underlying embryo resilience and support the future optimization of embryo production and cryopreservation strategies in cattle breeding under increasingly challenging environmental conditions.

## 1. Introduction

Heat stress is a major environmental factor limiting reproductive performance in livestock. Elevated ambient temperatures negatively affect fertility and pregnancy outcomes [1,2]. Even short periods of maternal hyperthermia around the time of fertilization and early embryogenesis can deteriorate blastocyst quality and increase the possibility of embryonic loss, with significant economic and welfare implications for animal production systems [3]. As global temperatures continue to rise, understanding how embryos respond to transient thermal stress has become increasingly important [1,2].

The sensitivity of bovine pre-implantation embryos to heat stress is strongly dependent on their developmental stage [4]. Embryos after cleavage are generally more susceptible to elevated temperatures than later stages, whereas thermotolerance increases as embryos develop toward the blastocyst stage [2,5]. This response has been attributed to key developmental events, including embryonic genome activation [6], changes in cellular metabolism [7], and the development of protective stress response mechanisms, such as developing thermotolerance by the accumulation of antioxidants [8]. Compacted morulae are able to maintain similar developmental rates under heat stress and thermoneutral conditions [9]. In contrast, exposure of embryos to heat stress during their early cleavage stages may exert effects that persist beyond the immediate stress period and influence later developmental competence [2,10].

At the cellular level, heat stress disrupts protein homeostasis, redox balance, and cell cycle regulation, triggering a range of adaptive responses aimed at maintaining cellular integrity [11,12]. The induction of molecular chaperones, commonly referred to as heat shock proteins (HSPs), plays a central role in protecting cells against protein misfolding and aggregation [13]. One of the first proteins to be induced following exposure to heat stress is HSP70 [2]. *HSPA14* has been identified as the major heat-inducible gene associated with the heat shock response and embryonic survival under thermal stress [14]. Beside *HSP70*, *HSP90* is one of the most studied heat shock proteins and has been implicated in heat stress-related cellular responses [15]. Although genes encoding heat shock proteins (HSPs) have been the most extensively studied, it has become increasingly evident that heat stress influences the expression of many other genes that are not directly involved in the heat shock response [16]. According to Feng et al. [17], heat stress significantly alters the expression of genes associated with oocyte developmental competence, cytoskeletal organization, mitochondrial function, and epigenetic regulation. The expression of DNA methyltransferase 3 alpha (*DNMT3A*) in blastocysts, which is responsible for epigenetic regulation of methylation, is significantly decreased after the exposure of oocytes to heat shock conditions. The downregulation of Glutathione Peroxidase 1 (*GPX1*), which plays a crucial role in protection against oxidative stress [18], was also observed. Placenta-Specific 8 (PLAC8), which is responsible for embryo–maternal interaction and placental development, was overexpressed compared to the heat-treated group [19]. The expression pattern of *DNMT3A* and *PLAC8* propose that although thermal shock reduces oocyte survival, the gametes that survive can retain the capacity to develop into healthy embryos [20]. On the contrary, De Barros and Paula-Lopes [11] established that heat stress disrupts epigenetic regulation in bovine embryos by hypomethylation of imprinted genes such as *H19* and insulin-like growth factor 2 (*IGF2R*), which are critical for early development.

MicroRNAs (miRNAs) are small noncoding RNAs that primarily regulate gene expression at the post-transcriptional level through degradation of target mRNAs or inhibition of their translation [21]. Sengar et al. [22] demonstrated that, out of 420 identified miRNAs, 65 showed differential expression under elevated temperature conditions, suggesting that miRNAs may play an important role in the regulation of cellular responses to heat stress. Heat stress conditions were associated with increased expression of miR-191-5p, while miR-378a-3p exhibited a decreasing expression tendency. Functional analysis revealed that several target genes of the selected miRNAs were associated with stress response and oxidative stress pathways. Furthermore, network analysis of differentially expressed miRNAs and their target genes indicated stress-mediated effects on cellular signaling mechanisms.

Previous studies investigating heat stress in bovine embryos have largely focused on acute responses, often reporting increased expression of stress-related genes and reduced developmental competence following exposure to elevated temperatures [23,24]. In contrast, results also suggest that moderate heat exposure during sensitive developmental windows may induce constant changes in gene expression and developmental patterns rather than transient stress responses alone [2,11,25]. These findings have contributed to diverging hypotheses regarding the biological significance of altered stress-related gene expression at later stages: while sustained activation may reflect unresolved cellular stress and impaired development, altered or reduced expression may indicate adaptive regulation or selective survival of embryos with enhanced stress resilience [2,26,27]. Exposure to sublethal stress during early development has been proposed to induce a form of developmental plasticity or conditioning, potentially enhancing resistance to later environmental or procedural stressors [28].

In the context of assisted reproductive technologies, cryopreservation represents a major challenge, imposing thermal, osmotic and oxidative stress that can affect blastocyst survival and molecular integrity after thawing [29]. Cryotolerance can vary among embryos, depending on a number of different factors [30,31]. According to Mori et al. [32] and Sakatani et al. [4] exposure of frozen–thawed blastocysts to heat stress impairs reproductive performance, although the extent to which early heat exposure modulates resistance of embryos to cryopreservation remains poorly understood.

Together, these observations highlight the complexity of embryonic responses to heat stress and the importance of considering both developmental timing and long-term consequences. The present study was therefore designed to examine how a controlled, physiologically relevant heat treatment applied during early morula stage influences subsequent embryonic development, molecular responses at the blastocyst stage, and resistance to additional stress imposed by cryopreservation [33,34].

## 2. Materials and Methods

### 2.1. Ovary Collection

The in vitro embryo production (IVEP) was carried out according to the protocol of IVF Limited T/A IVF Bioscience (Cornwall, UK) with minor modifications. Media were purchased from IVF Limited T/A IVF Bioscience media range, which included OPU, WASH, OIL, BO-HEPES-IVM, BO-IVF, BO-SEMPREP and BO-IVC unless otherwise specified.

Ovaries of *Bos taurus* were collected in phosphate buffer solution (PBS) (Thermo Fisher Scientific Inc., Waltham, MA, USA) in airtight nylon bags from the slaughterhouse of Tendon Kft. in Gyöngyös, Hungary. The transportation of the ovaries took approximately 45 min from the slaughterhouse to the laboratory in a polystyrene box. Before aspiration, extra tissues were removed using surgical scissors and the ovaries were kept in a beaker containing pre-warmed PBS (38.8 °C).

### 2.2. Oocyte Aspiration and In Vitro Maturation (IVM)

Follicular fluid was aspirated from 2–15 mm follicles using an 18G needle connected to a 5 mL three-part syringe and collected into 15 mL centrifuge tubes containing OPU medium + 10% fetal bovine serum (FBS) (Capricorn Scientific, Ebsdorfergrund, Germany). In a 9 cm Petri dish marked with a square grid pattern, 6–8 mL of OPU + 10% FBS was added, followed by the retrieval of the oocyte pellet from the bottom of the collection tube using a plastic transfer pipette. After searching under a stereo microscope, Grade 1 oocytes—according to the (International Embryo Technology Society, IETS)—with compact layers of cumulus cells and homogeneous cytoplasm were selected and then transferred to a 35 mm dish containing 2–3 mL of wash medium. Following the washing process, cumulus–oocyte complexes (COCSs) were subsequently transferred to cryogenic tubes containing 1 mL of BO-HEPES-IVM medium for in vitro maturation in a CO_2_ incubator at 38.8 °C for 21–24 h.

### 2.3. In Vitro Fertilization (IVF)

Fertilization was performed in a four-well culture dish (NUNC^®^, Thermo Fisher Scientific Inc., Rochester, NY, USA) in 500 µL of BO-IVF medium covered with 300 µL of OIL and pre-equilibrated in a gas mixture of 6% CO_2_ and 16% O_2_ for two hours at 38.8 °C. The mature oocytes were carefully transferred to the prepared BO-IVF medium, a maximum of one hour before fertilization without damaging their cumulus layer. During sperm preparation, the culture dish containing the COCs was returned to the incubator.

To avoid confounding effects arising from paternal variation, all in vitro fertilization procedures were performed using semen from the same bull. Straws containing semen were removed from liquid nitrogen, thawed in water at 37.8 °C for 20 s and emptied into a 15 mL centrifuge tube containing 4 mL of BO-SEMENPREP medium. The mixture was centrifuged for 5 min at 328× *g* in a heated centrifuge at 38.8 °C. After centrifugation, the supernatant was carefully removed, leaving 400 µL from the semen pellet. The washing step was repeated with 2 mL of BO-SEMENPREP medium and after removing the supernatant, sperm count and quality was analyzed.

Sperm concentration was calculated using a Makler counting chamber (Sefi Medical Instruments Ltd., Haifa, Israel). First, 5 µL of the semen sample was added to the chamber to evaluate the sample’s motility. Then, 10 µL of semen was mixed with 90 µL of water to dilute the sample and prevent motility. After suspending the mixture, 5 µL was placed on the cleaned chamber. After calculating the optimal amount, the final concentration of 2.0 × 10^6^ sperm/mL was added to the IVF wells and incubated for 16–19 h in a gas mixture of 6% CO_2_ and 16% O_2_ at 38.8 °C.

### 2.4. In Vitro Culture of Embryos (IVC)

The culturing was carried out in an IVFTech Freygen ALF six-chamber incubator (IVFtech, Klintehøj, Birkerød, Denmark). A 500 µL quantity of BO-IVC medium covered with mineral oil was equilibrated overnight in a gas mixture of 5.5–6.5% CO_2_ and 5.5–6.5% O_2_ at 38.8 °C. Presumptive zygotes were removed from the IVF dish into a 1 mL sterile Eppendorf tube containing 0.5 mL of preheated Wash medium and vortexed for 2 min at high speed to remove cumulus cells. The denuded zygotes were collected in a 35 mm Petri dish containing Wash medium and transferred into the four-well dishes for in vitro culture.

### 2.5. Heat Treatment

At 116 h post-insemination (hpi), the early morula stage embryos were selected and divided into equal groups according to the type of the experiment.

#### 2.5.1. Experiment A

Five morulas were placed in each BO-IVC medium droplets covered with mineral oil in 35 mm Petri dishes. During the experiment two chambers of the incubator were used, one for the control group and one for the treated groups. In the control group the culturing conditions described above were applied. In the treated group, the temperature was periodically raised from 38.8 °C to 40.0 °C by 0.1 °C every seven minutes. After reaching 40.0 °C, it was maintained for two hours and reduced back to the initial temperature. In the heat-stressed group, no gradual increase or decrease in temperature was applied. Instead, embryos were directly exposed to 40 °C for 2 h and subsequently returned to 38.8 °C. To determine differences in the expression profiles between the heat-treated and control groups, embryos were pooled within each group during sample collection before RNA isolation. The experiment was performed in 3 replicates. The number of embryos used is shown in Table 1.

#### 2.5.2. Experiment B

The same IVEP procedure as described for Experiment A was applied throughout Experiment B. The two experiments were conducted as independent IVEP sessions using separate batches of ovaries. Due to the high individual variability observed in the previous experiment, heat shock treatment was not applied in Experiment B. Embryos that reached the blastocyst stage were frozen applying slow freezing method. The media were originated from Minitube GmbH (Tiefenbach, Germany). Blastocysts were collected into BoviHold medium for washing and placed in BoviFreeze medium for eight minutes. Embryos were then loaded into 0.25 mL Minitube straws with the cryoprotectant solution and placed into the chamber of a pre-cooled EFT-3002 embryo freezer (Beltron Instruments, Longmont, CO, USA) at −6.5 °C. After two minutes, manual seeding took place using a copper bar to induce ice crystal formation. Ten minutes later, the instrument was started, and with controlled-rate slow freezing, the temperature in the chamber sank 0.5 °C/min, reaching −34.3 °C. Ten minutes later, the straws were taken out and directly placed into liquid nitrogen. Until thawing the embryos were kept in liquid nitrogen tanks at −196 °C.

For thawing, the straws were removed from liquid nitrogen and placed into tap water heated up to 37.8 °C for one minute. The straws were emptied into a 35 mm Petri dish, and after the removal of the cryoprotectant through washing in BoviHold medium, the embryos were placed into pre-equilibrated BO-IVC medium covered with mineral oil into the incubator for 24 h under the same conditions as during in vitro culture. Blastocyst survival and development were evaluated the following two days. The number of embryos used is shown in Table 2. The experiment was performed in 3 replicates.

### 2.6. Sample Collection

In Experiment A, the embryos that reached the blastocyst stage were washed in Dulbecco’s phosphate-buffered saline (DPBS) and collected into Eppendorf tubes containing 100 µL of lysis buffer solution (Thermo Fisher Scientific Inc., Waltham, MA, USA). In Experiment B, embryos were collected the same way one day after thawing. All embryos were stored at −70 °C until DNA extraction.

### 2.7. Molecular Analysis

#### RNA Isolation, cDNA Writing and qPCR

Embryos were placed in tubes containing 100 μL of RNA Aqueous Lysis Buffer Micro Kit (Applied Biosystems™, Thermo Fisher Scientific, Waltham, MA, USA) containing guanidinium thiocyanate. The embryo samples were stored at −80 °C until RNA isolation. RNA isolation was carried out with an RNAqueous™-Micro Total RNA Isolation Kit AM1931 (Applied Biosystems™). The isolated RNA was checked using a NanoDrop (ND-1000, UV-Vis Thermo Fisher Scientific, Waltham, MA, USA). For SYBR Green qPCR, the sample concentration must be 25 ng/μL, while for Advanced TaqMan qPCR, the sample concentration must be 5 ng/μL.

For mRNA marker analysis, cDNA was written from total RNA using a reverse transcription cDNA synthesis kit (High-Capacity cDNA Reverse Transcription Kit, Thermo Fisher Scientific, 145 Waltham, MA, USA). SYBR Green PCR master mix was used for qPCR analysis according to the manufacturer’s instructions (Thermo Fisher Scientific, 145 Waltham, MA, USA). The primers used for real-time PCR are shown in Table 3. For amplification, 7.5 μL of SYBR Green PCR master mix (Thermo Fisher Scientific, 145 Waltham, MA, USA), 0.75–0.75 μL of forward and reverse primers, 4.5 μL of Nuclease-Free Water (Thermo Fisher Scientific, 145 Waltham, MA, USA) and 1.5 μL of cDNA were measured per sample. The program set in the real-time PCR instrument included an initial 10 min denaturation step at 95 °C followed by 40 cycles of 95 °C for 15 s, 60 °C for 40 s and 68 °C for 20 s. Optical detection was performed at 68 °C.

Housekeeping genes (*EEF1A1*) were used to determine the expression of mRNA markers in the treated groups [20]. A control group was used as a reference. Each reaction was performed in triplicate. qPCR was performed on the collected embryos to analyze the expression profile of genes specific to embryonic development and heat shock proteins (*RPS15A-1*, *HSP90AA1-1*, *HSPB11-1*, *HSPA1A-1*, *GPX1-2*, *IGF2R-2*, *CCNB1-2*, *DNMT3A-2*, *PLAC8-2*) as shown in Table 3. There were 7–20 embryos in each group. The study was performed with three biological replicates. To determine differences in the expression profiles between the heat-treated and control groups, embryos were pooled within each group during sample collection before RNA isolation.

For miRNA amplification, advanced TaqMan buffered qPCR was used, for which samples were transcribed into cDNA using the Applied Biosystems TaqMan Advanced miRNA cDNA Synthesis Kit (catalog number: A28007, Thermo Fisher Scientific, Waltham, MA, USA). Due to the low concentration, the maximum sample volume (2 μL) was used without dilution during the experiment. The cDNA synthesis workflow consists of four steps: the first step is the poly(A) tailing reaction, followed by the adapter ligation reaction, followed by the reverse transcription (RT) reaction, and finally the miR-Amp reaction. All steps were performed according to the kit protocol, paying attention to the extra notes (e.g., 50% PEG 8000 reagent dissolution) and volumes. Until the real-time PCR was performed, the undiluted miR-Amp reaction product was stored at −80 °C until use.

TaqMan™ Fast Advanced Master mix (Applied Biosystems™) (catalog number: 4444963) for qPCR 4 μL Nuclease-Free Water (catalog number: AM9938), 10 μL TaqMan™ Fast Advanced Master Mix for qPCR (2×) (catalog number: 4444556), 1 μL TaqMan Advanced miRNA Assay/miRNA probe (20×) (catalog number: A25576) were measured and multiplied by the number of samples for each primer (Table 4) [22,35,36,37]. Then, 5 μL cDNA template was added to each primer Master mix so that the final concentration per well was 0.001–100 ng/well. The used advanced miRNA primers are shown in Table 4.

Additionally, 4 μL of RNase-free water was added as well as a negative control. For each biological replicate, every sample was analyzed in technical triplicate for each tested miRNA. miR-92 was used as an internal control (as housekeeping gene).

### 2.8. Apoptosis and Necrosis Staining

Embryo apoptosis and necrosis staining was performed using a commercially available FITC–Annexin V apoptosis and necrosis detection kit (PromoKine, Heidelberg, Germany; PK-CA707-30018). Briefly, a 1× binding buffer was prepared by diluting 100 μL of 5× binding buffer in 400 μL of sterile embryo-quality water. The staining solution consisted of 100 μL of 1× binding buffer supplemented with 5 μL FITC–Annexin V, 5 μL Ethidium Homodimer III, and 5 μL Hoechst 33342. The staining mixture was gently mixed and protected from light using aluminum foil. Embryos were incubated in 50 μL drops of staining solution for 15 min at room temperature in the dark. Following incubation, 200 μL of 1× binding buffer was added for washing. Hoechst 33342 was used for total nuclear staining, FITC–Annexin V for the detection of phosphatidylserine externalization during apoptosis, and Ethidium Homodimer III for the identification of necrotic cells with compromised membrane integrity. Fluorescence imaging was performed using an ImageXpress^®^ Pico Automated Cell Imaging System (Sunnyvale, CA, USA) with DAPI/Hoechst, FITC, and Texas Red filter settings. For apoptosis and necrosis analysis, 16 control and 17 heat-treated embryos were used.

### 2.9. Immunofluorescence Staining

For immunostaining, embryos were first washed in 1× PBS containing 0.1% bovine serum albumin (BSA) for 1 min at room temperature and subsequently fixed in 4% paraformaldehyde supplemented with 0.1% BSA for 10 min. After fixation, embryos were washed in 0.01% BSA-PBS and stored at 4 °C in the dark until further processing. Permeabilization and blocking were performed for 40 min at room temperature in blocking solution. Embryos were then incubated overnight at 4 °C in the dark with primary antibodies diluted in 0.1% BSA-PBS, including rabbit monoclonal anti-CDX2 (1:100; Abcam, Cambridge, UK, ab76541) and rabbit monoclonal anti-OCT4 (1:100; Abcam, ab181557). Following three washing steps in 0.01% BSA-PBS, embryos were incubated for 1 h at 37 °C in the dark with goat anti-rabbit secondary antibody conjugated with Abberior STAR RED (1:200; Abberior/Unicam). Nuclear counterstaining was performed using TO-PRO-3 iodide (1:500; Thermo Fisher Scientific) for 15 min at room temperature. After additional washing steps, embryos were mounted in antifade mounting medium (Abberior Mount Liquid Antifade, Unicam), covered with coverslips, stored at 4 °C in the dark, and visualized using confocal microscopy (TCS SP8, Leica Microsystems IR GmbH Wetzlar, Hessen, Germany). In total, 15 control and 13 heat-treated embryos were subjected to immunofluorescence staining.

### 2.10. Statistical Analysis

A Generalized Linear Mixed Model (GLMM) with a binomial family and a logit link function was used to assess the effect of different treatments in the experimental groups. The models were fitted by maximum likelihood (Laplace approximation) using the glmer() function from the lme4 package in R (R version 4.6.0). In case of blastocyst formation, the treatment was included as the fixed effect, while experiment and culture droplet were included as random effects to account for the non-independence of embryos cultured within the same droplet and across separate experimental runs. For post-thaw survival, the treatment was the fixed effect and the experiment was the random effect. To investigate specific differences between the groups, pairwise comparisons were performed (Tukey’s HSD) using the glht() function from the multcomp package. The RT-qPCR results were processed using the Eppendorf RealPlex program. The obtained data were analyzed with MultiD GenEx qPCR data analysis software (http://www.multid.se, version 7.0).

The Norm Finder tool, integrated within the GenEx software package (version 7.0), was used to identify the most stable reference genes for normalization in qPCR. According to this analysis, EEF1A1 and miR-92a-3p as an internal control were applied.

The RT-qPCR data were analyzed according to the 2^−ΔΔCT^ method; they expressed the fold change of each selected gene within experimental groups. Data were normalized against the control (expression was set to 1). The fold change for each gene was subjected to one-way ANOVA followed by Student’s *t*-test [38].

The SOM (self-organizing map) hierarchical clustering function was used to create groups to illustrate the difference between expressions and the correlations between samples. A small self-organizing map (SOM) can be used to force classification into a defined small number of groups based on expression similarities. The SOM algorithm projects the multidimensional mRNA/miRNA expression data into this two-dimensional space, so that samples with similar mRNA/miRNA expression profiles are placed closer together. Therefore, the important information in the figure is the relative position of the samples. Samples close together have more similar mRNA/miRNA expression profiles, whereas samples positioned farther apart exhibit greater differences in their expression patterns.

Values below * *p* < 0.05 were considered significant results.

## 3. Results

Numerous data from the literature confirm that gene expression patterns are sensitive to the developmental stage of the embryo [39,40,41,42]. Since embryonic development is a continuously evolving process, it is very difficult to identify which changes are actually influenced by the treatment. In our study, we therefore ensured that measurements were taken from blastocyst-stage embryos, and we evaluated expression levels in each experiment and for each treatment relative to the values of the respective control embryos. The control and heat-treated groups from the same replication were compared, as the gene expression of control embryos from different replicates showed differences between experiments, probably due to different environmental conditions of the donor cows and individual differences.

### 3.1. Blastocyst Rates

#### 3.1.1. Experiment A

A total of 270 early morula-stage embryos were included in Experiment A. Embryos were equally distributed among the control, heat-treated, and heat-stressed groups (n = 90 per group) across three biological replicates. The blastocyst rates of each replicate are shown in Table 5.

**Figure 1:**
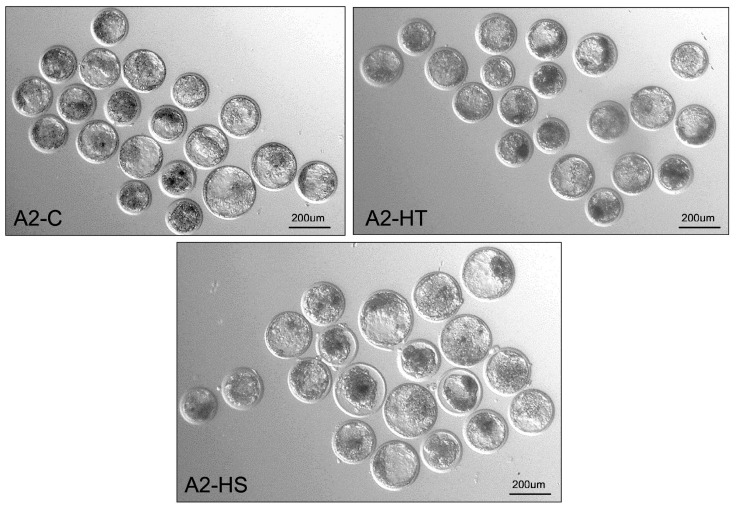
Blastocysts in each group of Experiment A2.

The embryos that reached the blastocyst stage in Experiment A2 are shown in Figure 1.

**Figure 2:**
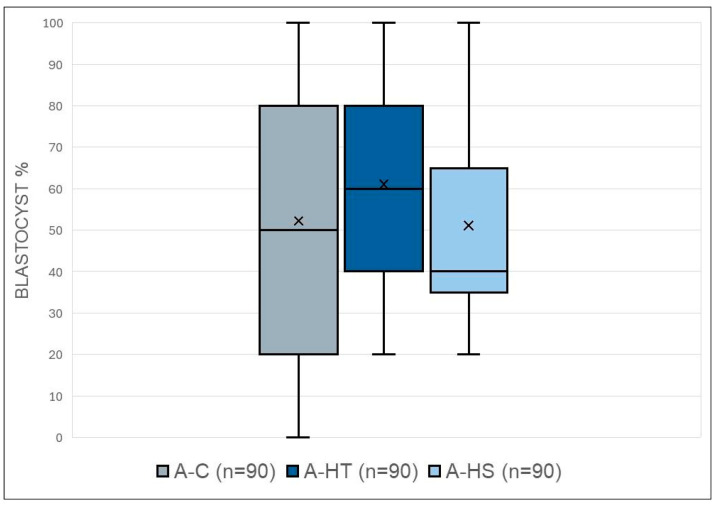
Average blastocyst rates of Experiment A.

The average blastocyst rates from all three replicates are shown in Figure 2.

The pairwise comparisons between the groups indicated no statistically significant differences in blastocyst formation rates between any of the treatment groups. Specifically, HT group to the control group: *p* = 0.464; HS group to the control group: *p* = 0.988; and HS group to the HT group: *p* = 0.381. Model-estimated blastocyst probabilities were 50.3% (control), 59.9% (HT) and 49.2% (HS). Most of the non-treatment variability was due to differences between experiments (random intercept SD = 0.41), and, to a lesser extent, due to droplet (SD = 0.29).

#### 3.1.2. Experiment B

Overall, 64 blastocysts were subjected to cryopreservation in the second experiment, consisting of 29 control and 35 heat-treated embryos.

Variation in post-thaw survival rates was evident among individual experimental runs in both control and heat-treated groups. The post-thawing survival rates are shown on Table 6.

**Figure 3:**
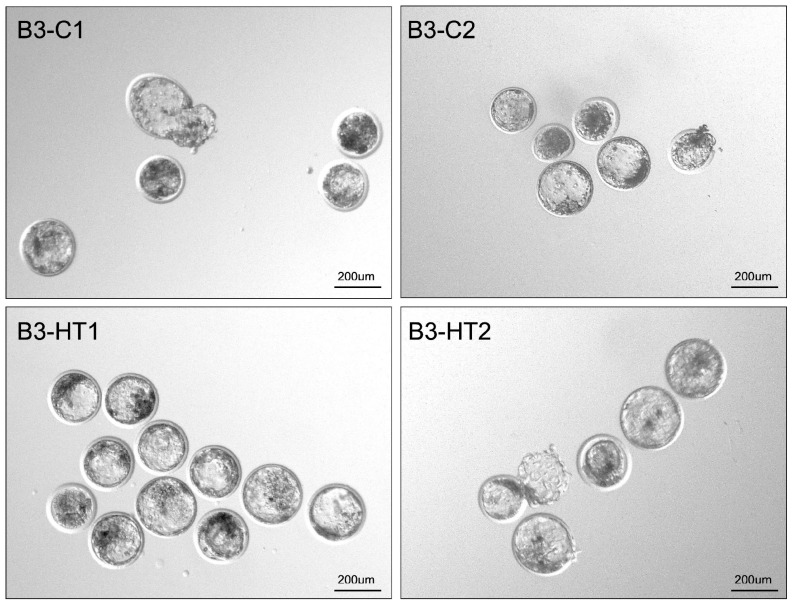
Control and heat-treated embryos 24 h after thawing in Experiment B3.

Embryos of the control and heat-treated groups in Experiment B3 24 h after thawing are shown in Figure 3.

**Figure 4:**
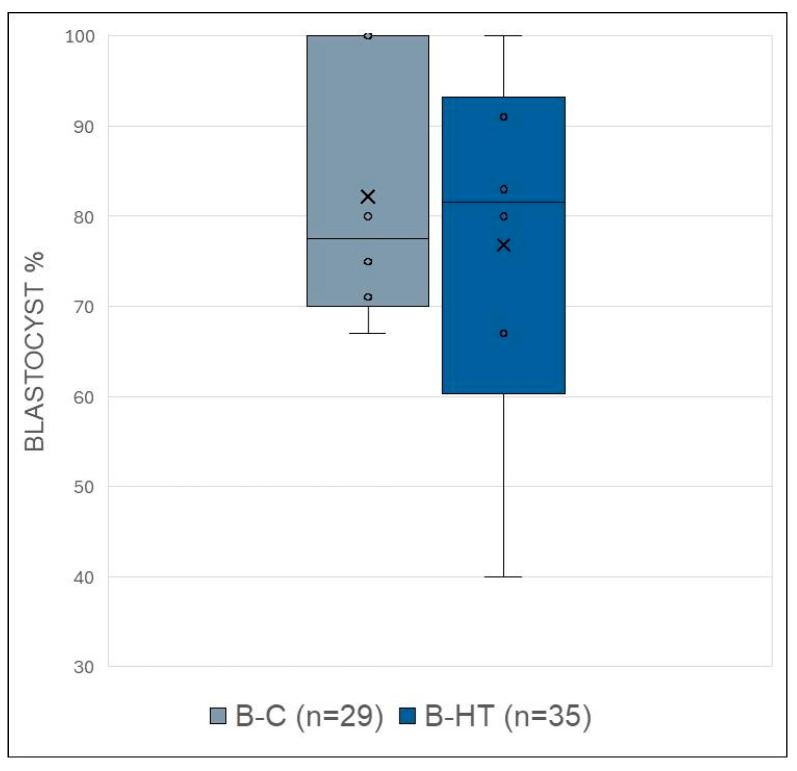
Average survival rates of the thawed embryos.

Control embryos presented survival rates ranging from 73% to 100% while treated embryos varied between 60 and 95%. The average survival rates of all three replicates are shown in Figure 4.

The treatment did not significantly affect post-thaw survival (*p* = 0.957); model-estimated survival was 79.5% (control) versus 80.0% (HT). Between-experiment variation was small (random intercept SD = 0.16).

### 3.2. Gene Expression Patterns

#### 3.2.1. Experiment A

In Experiment A (A_C; A_HT and A_HS), the expression levels of numerous genes that are crucial for embryonic development and are associated with heat stress were determined.

**Figure 5:**
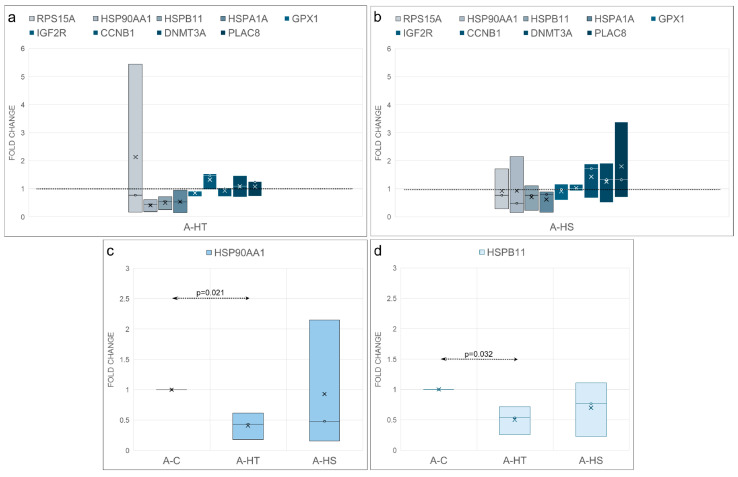
(**a**) Relative expression levels of the examined genes in the heat-treated group. (**b**) Relative expression levels of the examined genes in the heat-stressed group. (**c**) Relative expression of the *HSP90AA1-1* gene (*p* = 0.021). (**d**) Relative expression of the *HSPB11-1* gene (*p* = 0.032).

Relative expression levels of the examined genes in both treatment groups (HT—heat treatment; HS—heat stress) relative to the control group are shown in Figure 5a,b. The values were normalized to the *EEF1A1* reference gene, where the control group value was 1. Figure 5c,d highlight the altered expression of the *HSP90AA1* and *HSPB11* genes in response to heat treatment and heat stress, respectively. In both cases, heat treatment resulted in a significant decrease in expression level of the *HSP90AA1-1* gene (*p* = 0.021) and *HSPB11-1* (*p* = 0.032). The gene expression responses of embryos to heat stress are so unique that the standard deviation was very high for the values of the examined groups.

**Figure 6:**
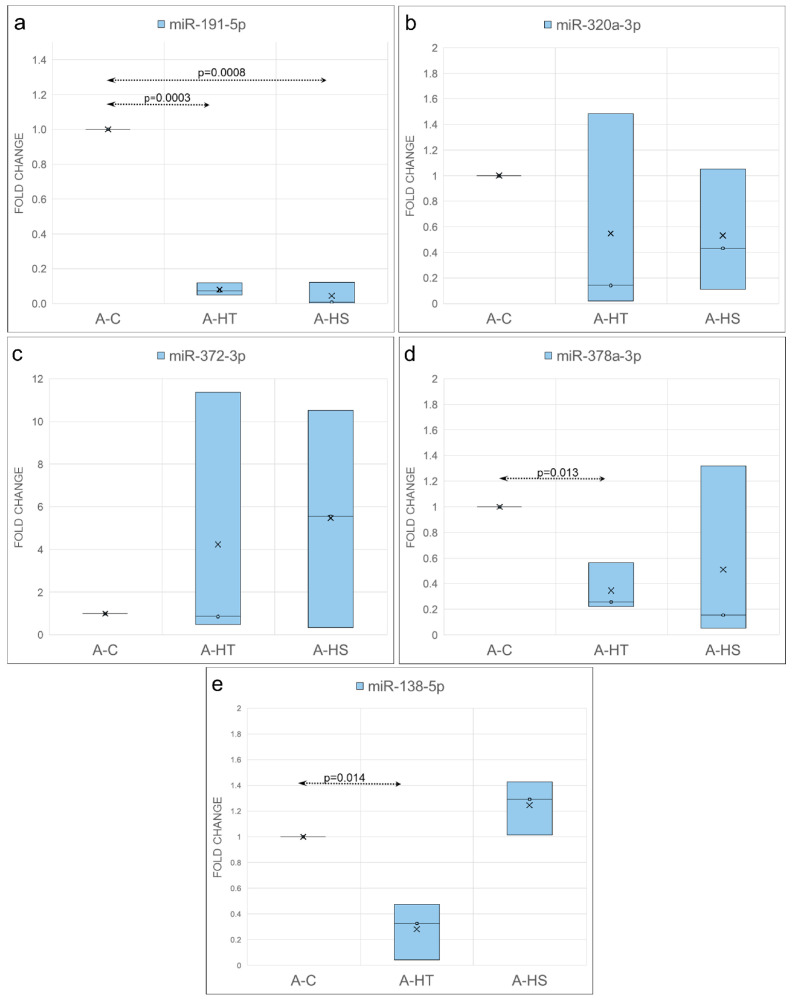
Relative expression levels of the examined microRNAs: (**a**) Relative expression levels of miR-191-5p. (**b**) Relative expression levels of miR-320a-3p. (**c**) Relative expression levels of miR-372-3p. (**d**) Relative expression levels of miR-378a-3p. (**e**) Relative expression levels of miR-138-5p.

The expression levels of several miRNAs in the samples from Experiment A was also measured and shown in Figure 6. It is increasingly accepted that miRNAs play a key role in regulating gene expression at the post-translational level. Figure 6. shows the relative expression levels of the miR-191-5p, miR-372-3p, miR-378a-3p, miR-138-5p and miR-320a-3p miRNAs in both treatment groups compared to the control. Relative expression levels were normalized to the miR-92a-3p reference gene, where the control group value is 1.

Among these miRNAs a significant decrease in expression levels for miR-191-5p were observed in both treatment groups compared to the control (*p* < 0.001 in the heat-treated group and *p* < 0.001 in the heat-stressed group) (Figure 6a), while for miR-378a-3p (*p* = 0.013) (Figure 6d) and miR138-5p (*p* = 0.015) (Figure 6e), a significant decrease was observed in the heat-conditioned group compared to the control group.

**Figure 7:**
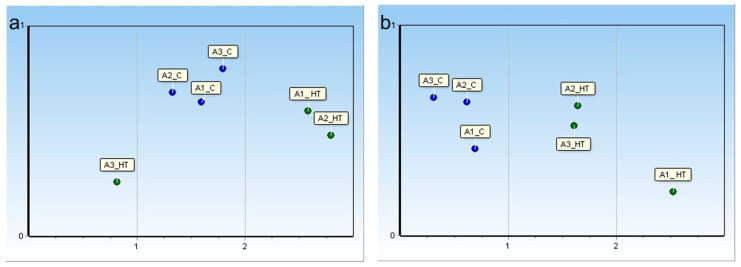
Kohonen clustering shows the clustering of the three treatment groups based on mRNA (**a**) and miRNA (**b**) expression profiles, where control groups are indicated in blue and heat-treated groups in green.

In addition, Kohonen’s self-organizing map (SOM) was used to identify clusters exhibiting similar expression patterns as an exploratory visualization of expression similarity among samples. As shown in Figure 7, the controls always appear together; in Experiment A, the changes in marker 7(a)’s expressions in response to treatments were real, but the effects of heat treatment cannot be clearly distinguished, likely due to individual reactions. The miRNA expression responses in Figure 7b are not as clear-cut; there are groups (A2_HT; A3_HT) that did not differ from the controls, while there were groups (A1_HT; A2_HT) where the effect of heat treatment was confirmed. Heat-stressed groups were excluded from graphical representation because of the high degree of individual variability observed among the samples.

#### 3.2.2. Experiment B

**Figure 8:**
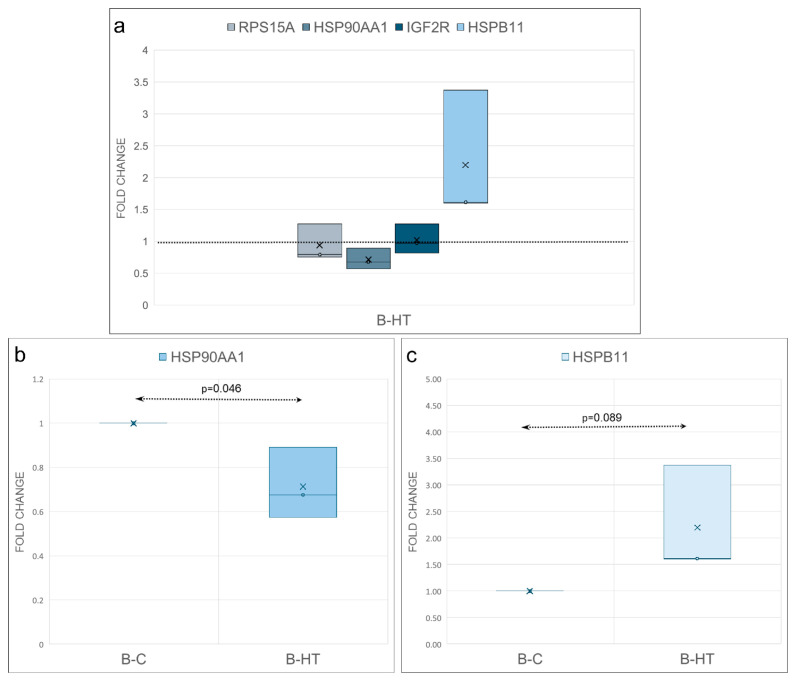
(**a**) The relative expression levels of the examined genes. (**b**) Relative expression levels of *HSP90AA1* gene. (**c**) Relative expression levels of *HSPB11* gene.

In Experiment B (B_C and B_HT), only heat conditioning was applied, as the embryos’ response to heat stress was too specific. Relative expression of the examined genes is shown in Figure 8. The relative expression levels were normalized to the *EEF1A1* reference gene, with the control group set to 1. Figure 8a shows that *HSP90AA1* expression decreased significantly in response to heat treatment (*p* = 0.046), while the expression of the other markers remained similar to the control group. The decrease in *HSP90AA1* expression is significant even among these experimental parameters (Figure 8b), and HSPB11 showed the same downward tendency as in Experiment A but did not reach significance (*p* = 0.089) owing to large individual variation (Figure 8c).

**Figure 9:**
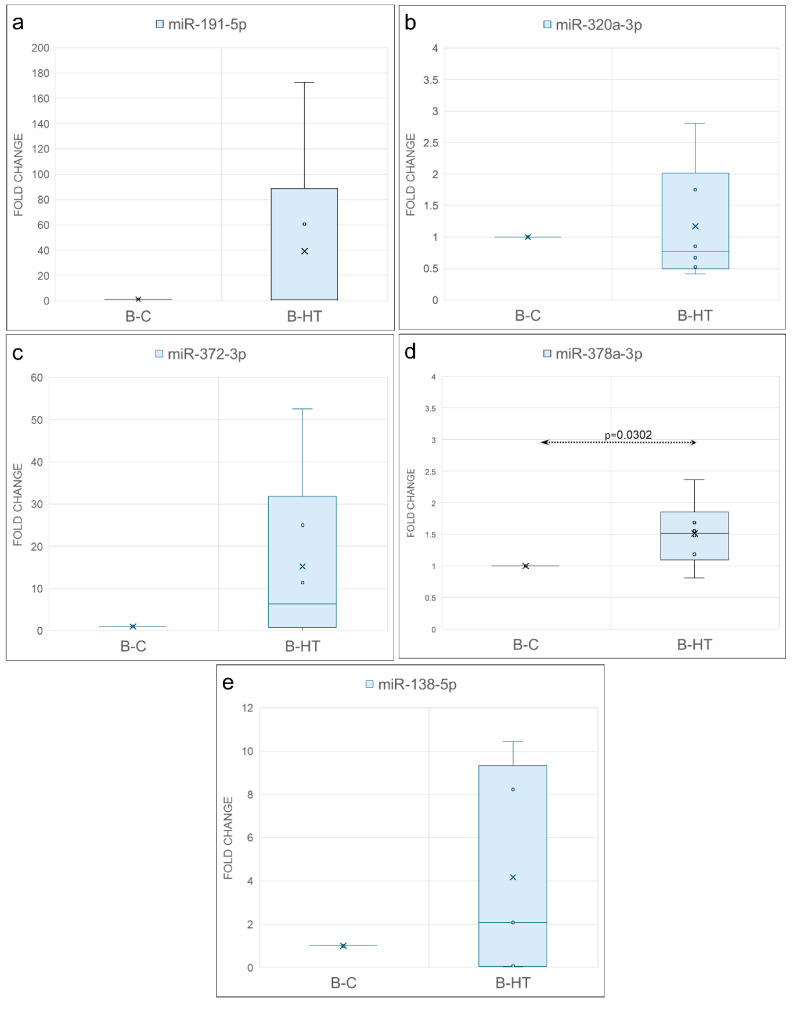
Relative expression levels of the examined miRNAs in Experiment B: (**a**) Relative expression levels of miR-191a-5p; (**b**) miR-320a-3p; (**c**) miR-372-3p; (**d**) miR-378-3p; (**e**) miR-138-5p.

The miRNA profile in Experiment B was highly variable even within the heat-conditioned group in the treated embryos because of freezing, resulting in very large standard deviations (Figure 9). The values were normalized to the miR-92a-3p reference gene, where the control group value is 1. Only the expression of miR-378a-3p was stable, showing a significant increase following heat treatment compared to the control group as shown in Figure 9d (*p* = 0.03). Genes that were also important in the previous experiment are highlighted in Figure 9, but no significant difference was observed.

**Figure 10:**
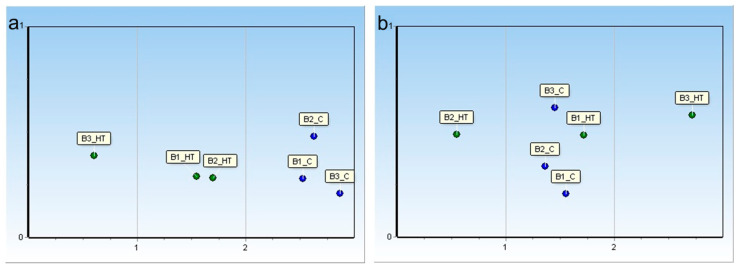
Kohonen clustering, showing the clustering of the two treatment groups in Experiment B based on mRNA (**a**) and miRNA (**b**) expression profiles, where control groups are indicated in blue and heat-treated groups in green.

In Experiment B, Kohonen clustering was also performed, with the controls appearing together in both marker tests as shown in Figure 10. Among the treated groups, there was one in each where the effect of heat treatment was not apparent. In the case of markers, the B2_HT (Figure 10a) group, and in the case of miRNAs, the B1_HT (Figure 10b) group were similar to the controls, while the effect of the treatment was evident in the other groups.

**Figure 11:**
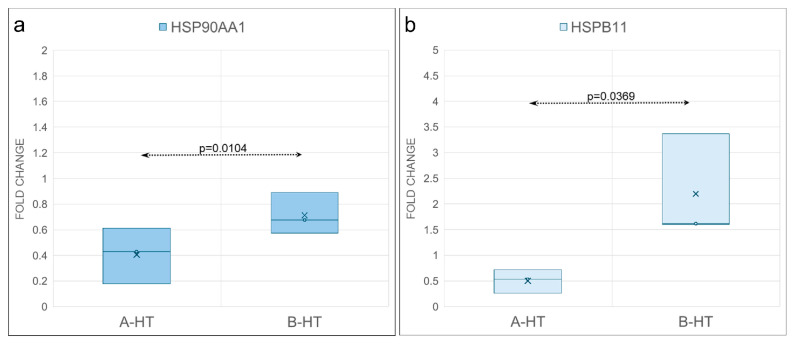
(**a**) Expression levels of the *HSP90AA1* and (**b**) *HSPB11* genes in the HT groups in Experiments A and B.

However, when examining the expression of the *HSP90AA1* and *HSPB11* genes in the HT groups in Experiments A and B (where the values for both groups were evaluated relative to their own controls), it can be seen that expression increased for both markers following freezing, which represents a significant increase compared to Experiment A (Figure 11) (*HSP90AA1* (*p* = 0.01) and *HSPB11-1* (*p* = 0.036)).

### 3.3. Apoptosis, Necrosis and Immunofluorescence Staining

**Figure 12:**
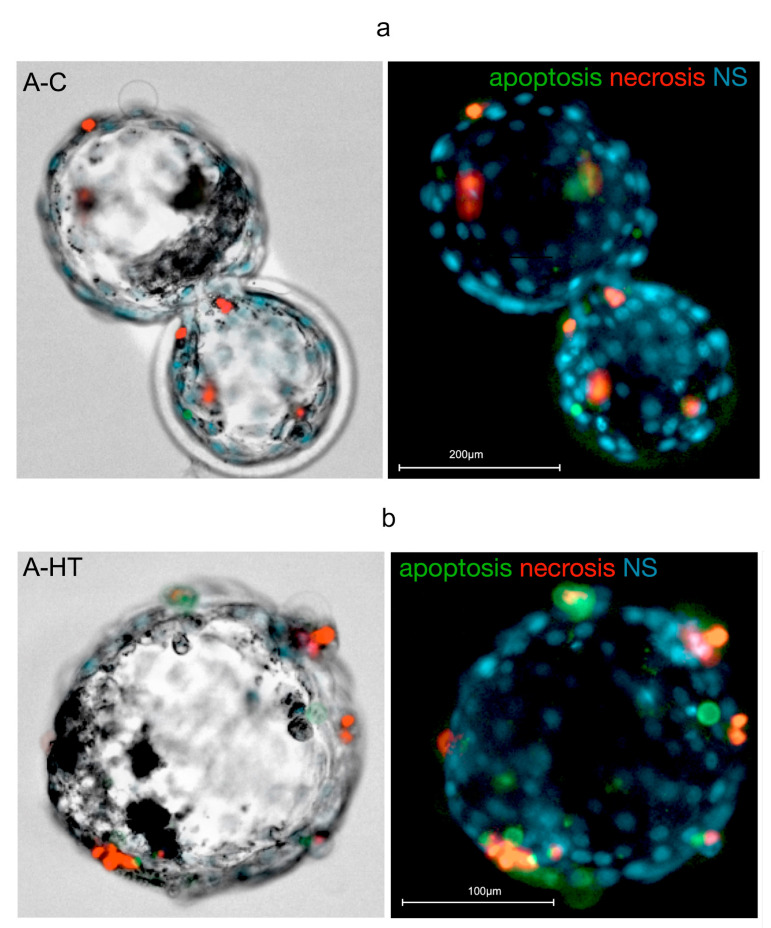
Apoptosis and necrosis staining of (**a**) control embryo (C) and (**b**) heat-treated embryo (HT). Green color shows apoptotic cells; red color shows necrotic cells and blue color shows nuclei.

**Figure 13:**
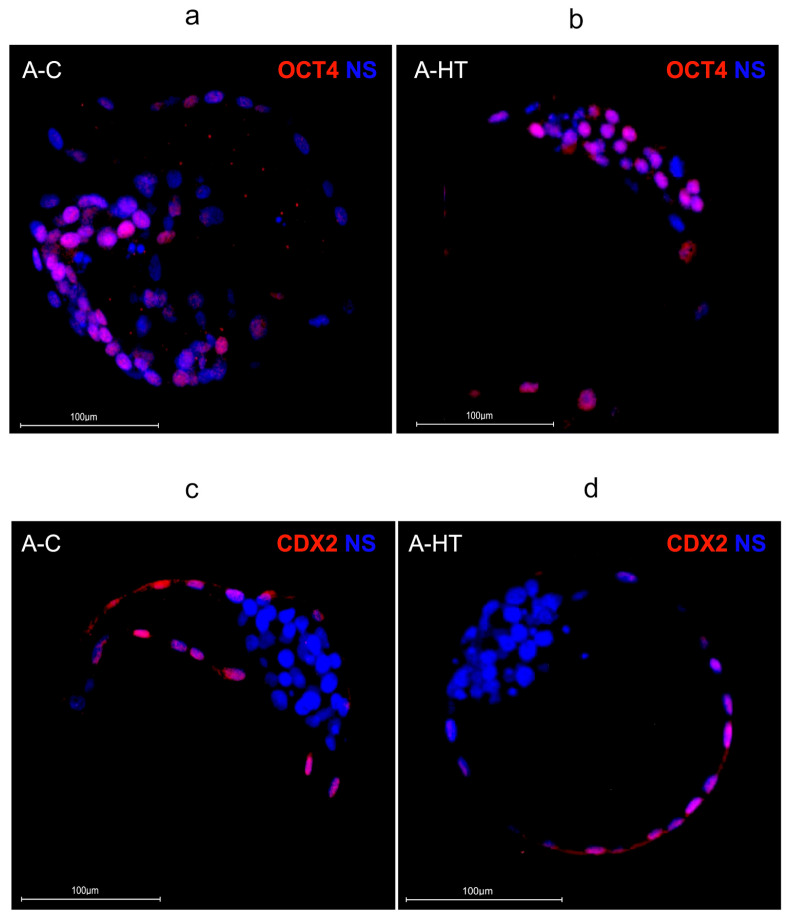
OCT4 staining of (**a**) control embryos (C) and (**b**) heat-treated embryos (HT). CDX2 staining of (**c**) control embryos and (**d**) heat-treated embryos. Red color shows CDX2-positive cells and blue color shows OCT4-positive cells.

Representative apoptosis/necrosis staining (Figure 12) and immunofluorescence images (Figure 13) are presented to qualitatively evaluate embryo morphology and marker localization following treatment. Apoptosis/necrosis staining showed the presence of viable embryos in both the control and heat-treated groups, with no apparent qualitative differences. Immunofluorescence staining demonstrated *CDX2* localization in the trophectoderm and *OCT4* expression in the inner cell mass in all embryos.

## 4. Discussion

Bovine embryos are highly sensitive to elevated temperatures, particularly during the early stages of development, when cellular and molecular processes are vulnerable to environmental stress [9]. In this context, the present study evaluated whether controlled heat treatment can modulate embryonic developmental competence and stress response.

A controlled heat-treatment protocol for bovine embryos was described, in which thermotolerance is induced through the gradual elevation, maintenance, and subsequent reduction of temperature from 38.8 °C to 40.0 °C and back to 38.8 °C.

The selection of the optimal time point for applying heat treatment was guided by the stage-dependent sensitivity of bovine embryos, as thermotolerance increases after embryonic genome activation, such that morula-stage embryos are markedly less susceptible than earlier cleavage-stage embryos [4]. Based on this developmental shift, the early morula stage was established for heat treatment, as it represents a developmental window characterized by increased resistance to heat stress and an enhanced capacity to mount transcriptional stress responses [43].

Heat stress is generally associated with reduced blastocyst formation rates in bovine embryos [3,44]. However, consistent with the findings of Oliveira et al. [25], heat treatment did not significantly decrease blastocyst development in the present study, suggesting that the applied thermal exposure represented a mild stress condition rather than a severely detrimental one. Similar observations regarding stress adaptation and acquisition of thermotolerance following controlled heat exposure have also been described previously in bovine embryos [25,45]. Furthermore, post-thaw blastocyst survival was not significantly affected by heat treatment, indicating that the applied thermal exposure did not impair embryonic survival following cryopreservation. These findings may suggest that the treated embryos retained their developmental competence despite exposure to combined thermal and cryogenic stress conditions.

Contrary to several previous studies reporting increased or unchanged HSP90AA1 expression following heat stress [12,43], the present study observed decreased expression, which may reflect differences in developmental stage, heat stress duration, embryo quality, or experimental conditions. The significantly lower expression of *HSP90AA1* observed in both heat-treated and cryopreserved embryos may indicate that the applied thermal exposure did not induce severe cellular stress requiring strong activation of stress response pathways. Heat shock proteins are known to play essential cytoprotective roles during early embryonic development through maintenance of protein folding, cellular homeostasis, and genome stability under stress conditions. In agreement with the importance of this pathway, Zhao et al. [46] demonstrated that inhibition of *HSP90AA1* significantly reduced cleavage and blastocyst formation rates while increasing embryo fragmentation and decreasing blastocyst quality. Furthermore, inhibition of *HSP90AA1* resulted in lower total cell numbers, reduced trophectoderm (TE) and inner cell mass (ICM) cell numbers, as well as alterations in epigenetic markers associated with genome stability and DNA repair processes. Despite the reduced *HSP90AA1* expression observed in the present study, neither blastocyst development nor post-thaw survival was significantly impaired. Since both heat stress and cryopreservation are generally associated with oxidative stress, protein denaturation, cytoskeletal disruption, and DNA damage, the reduced expression of *HSP90AA1* may indicate a lower requirement for activation of stress response mechanisms under the applied experimental conditions. In addition to *HSP90AA1*, the expression of HSPB11 was also significantly lower in Experiment A and showed a similar tendency in Experiment B, whereas the latter was not statistically significant due to large individual variation. Although the specific role of *HSPB11* during bovine pre-implantation development remains poorly characterized, previous studies suggested that HSPB11 may function as a cytoprotective factor through stabilization of mitochondrial membranes and activation of *HSP90*- and *PI3K*–Akt-associated survival pathways [47]. The lower expression of *HSPB11* observed may therefore further support the hypothesis that the applied treatment conditions did not induce severe cellular stress responses.

In the present study, the expression levels of *HSP90AA1* and *HSPB11* were significantly increased in heat-treated cryopreserved embryos compared to embryos exposed only to heat treatment. This may suggest that the combination of thermal exposure and cryopreservation induced a more pronounced cellular stress response than heat treatment alone. Nevertheless, further investigation is required to clarify the molecular mechanisms underlying the adaptive responses observed following combined thermal exposure and cryopreservation, as the stress response-related molecular changes induced by controlled heat treatment prior to cryopreservation have not yet been extensively characterized in bovine embryos.

miRNAs play a key role in the post-transcriptional regulation of gene expression. To confirm the molecular alterations induced by thermal treatment, gene expression analyses were performed using development- and heat stress-related markers selected based on previous literature data [22,35]. The applied thermal exposure did not affect the expression of development-specific markers in any of the experiments. In contrast, the expression of heat stress-associated markers decreased under heat conditioning conditions, whereas significant upregulation was observed under heat stress conditions. In Experiment B, cryopreservation appeared to act as an additional stress factor, as increased expression levels were detected for all examined markers and miRNAs following freezing. Potential relationships among markers showing significant expression changes in response to thermal treatment were further investigated. It was hypothesized that these markers may contribute to the development of stress resistance through regulatory signaling pathways.

To evaluate this hypothesis, publicly available databases were utilized. Analysis of the miRBase database [37] revealed that miR-378a-3p, the miRNA showing significant expression changes in the present study, possesses a predicted target site within the 3′ untranslated region (3′ UTR) of *AHSA1* and may therefore play a role in the regulation of its expression. *AHSA1* functions as a co-chaperone and activator of *HSP90AA1* [48,49].

**Figure 14:**
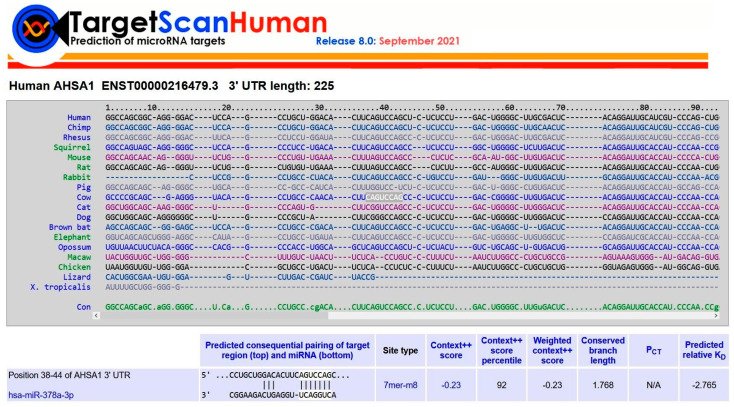
miR-378a-3p predicted target in TargetScan (based on Agarwal et al. and McGeary et al. [48,49]).

The latest version of TargetScan (v8.0; targetscan.org) (shown in Figure 14) was used for miRNA target prediction analysis. TargetScan is a freely accessible database widely used for the identification of miRNA target sites and investigation of the regulatory roles of microRNAs in protein expression [48,49].

It is hypothesized that miR-378a may play a protective role in the embryonic response to stress conditions. The decreased expression observed following heat conditioning may indicate that embryos with improved adaptive capacity require less activation of stress-associated regulatory mechanisms. In contrast, the elevated expression detected following combined heat treatment and cryogenic stress may reflect the activation of compensatory mechanisms counteracting cellular stress. Furthermore, hsa-mir-378a expression tends to decrease in several types of cancers, including bladder cancer (BLCA), breast cancer (BRCA), colon adenocarcinoma (COAD), and lung adenocarcinoma (LUAD), which may suggest a tumor suppressor role or involvement in the progression of these diseases [36].

Further studies are required to better characterize the molecular mechanisms underlying the adaptive responses induced by controlled heat exposure prior to cryopreservation. Since the regulatory roles of several stress-associated miRNAs identified in the present study remain poorly characterized during bovine pre-implantation development, additional investigations focusing on miRNA-mediated signaling pathways and miRNA–mRNA interactions may provide further insight into embryonic stress adaptation mechanisms. Although the molecular changes detected in the present study were not reflected in improved blastocyst development or cryosurvival, they indicate that controlled heat exposure was sufficient to trigger measurable cellular responses. Future studies should investigate whether combining this approach with compounds that enhance cellular stress resistance, such as coagulansin-A, could potentiate these molecular responses and ultimately improve thermotolerance, as suggested by previous studies [50]. Moreover, prolonged in vitro culture experiments performed under different temperature conditions simulating thermal stress may help to evaluate the longer-term effects of combined thermal exposure and cryopreservation on embryonic developmental competence, survival capacity, thermotolerance, and adaptive responses.

## 5. Conclusions

The present study demonstrates that a controlled and physiologically relevant heat treatment applied during the early morula stage did not impair blastocyst development or post-thaw survival of bovine embryos. Although no significant differences were observed in developmental competence, important alterations in stress-related molecular markers were detected. In particular, the reduced expression of *HSP90AA1* and *HSPB11* following heat treatment is consistent with molecular responses to thermal exposure under conditions that were not severely detrimental to embryonic development. Furthermore, significant changes in the expression of several miRNAs, especially miR-378a-3p and miR-191-5p, suggest that post-transcriptional regulatory mechanisms may contribute the molecular response of embryos to thermal stress. Cryopreservation induced additional molecular responses, reflected by increased expression of stress-associated markers in frozen–thawed embryos, highlighting the complexity of combined thermal and cryogenic stress. Overall, the findings suggest that controlled heat conditioning at the morula stage induces molecular responses without compromising embryonic viability. However, further investigations are required to clarify the molecular pathways involved in heat-induced embryonic adaptation and to determine the long-term developmental consequences of combined thermal exposure and cryopreservation in bovine embryos.

## Figures and Tables

**Table 1:** The number of embryos assessed in each replicate of Experiment A.

Replicate	Group Type	Number of Embryos per Treatment Group	Number of Embryos for Molecular Analysis
A1	C	20	10
	HT	20	10
	HS	20	7
A2	C	40	20
	HT	40	20
	HS	40	20
A3	C	30	12
	HT	30	12
	HS	30	12

C: control; HT: heat-treated (gradual 38.8→40.0→38.8 °C); HS: heat-stressed (direct 40.0 °C for 2 h).

**Table 2:** The number of embryos used in each replicate of Experiment B.

Replicate	Group	Number of Cryopreserved Embryos	Number of Embryos for Molecular Analysis
B1	C	7	7
	HT	9	7
B2	C	11	8
	HT	10	6
B3	C	11	8
	HT	16	15

C: control; HT: heat-treated (gradual 38.8→40.0→38.8 °C).

**Table 3:** Used forward and reverse mRNA primers [20].

Name of mRNAs	Forward Primer	Reverse Primer	Product Size
EEF1A1 #	CCCCAGGACACAGAGACTTC	ATTCACCAACACCAGCAGCA	93
RPS15A	TCAGCCCTAGATTTGATGTGC	TTCCCTCCTGTATGTTTTCGTC	148
HSP90AA1	CTGGAAGGAGACGACGACAC	ACACACTGGAGGGAATGGAG	103
HSPB11	AGAGGAGGCGGAGGACGG	GGTGATAGGGCACGAAGCAA	117
HSPA1A	GGACCTGCTGTTGCTGGAC	TTCGTGGGGATGGTGGAGTT	103
GPX1	GAAAAGTGCGAGGTGAATGG	GAGAGCAGTGGCGTCGTC	93
IGF2R	GGACTACAGGCATCAGGACG	AACACGAAGGGGAACACACA	105
CCNB1	ATACTCCCTCTCCAAGCCCC	ATCCGCTCCGTCTTCTGC	122
DNMT3A	GAAGGAGCATTTGGGAACAG	GTTATTGCGTGAGCCTGGAT	117
PLAC8	GTTTCACAGCCAGGTTACAGC	AGAGCCCCACAGAGACAGAT	104

# internal control.

**Table 4:** Used advanced miRNA primers (Catalog Number: A25576) [22,35,36,37].

Name of miRNAs	ID Number	miRNA Sequency
hsa-miR-92a-3p #	477827_mir	UAUUGCACUUGUCCCGGCCUGU
hsa-miR-191-5p	477952_mir	CAACGGAAUCCCAAAAGCAGCUG
hsa-miR-320a-3p	478594_mir	AAAAGCUGGGUUGAGAGGGCGA
hsa-miR-372-3P	478071_mir	AAAGUGCUGCGACAUUUGAGCGU
hsa-miR-378a-3p	478349_mir	ACUGGACUUGGAGUCAGAAGGC
hsa-miR-138-5p	477905_mir	AGCUGGUGUUGUGAAUCAGGCCG

# internal control.

**Table 5:** Blastocyst rates in each replicate.

Replicate	Group	Blastocyst Rate (%)
A1	C	50 ± 25.8
	HT	50 ± 20.0
	HS	35 ± 10.0
A2	C	62.5 ± 32.8
	HT	72.5 ± 18.3
	HS	67.5 ± 26.0
A3	C	40 ± 28.3
	HT	53.3 ± 16.4
	HS	40 ± 21.9

±: the standard deviation across the culture droplets within each replicate.

**Table 6:** Survival rates of control and heat-treated groups after thawing.

Replicate	Group	Survival Rate (%)
B1	C	100
	HT	75
B2	C	73
	HT	60
B3	C	73
	HT	95

## Data Availability

The corresponding author will provide the data upon request.

## Abbreviations

The following abbreviations are used in this manuscript:

AHSA1	activator of HSP90 ATPase activity 1
Akt	protein kinase B
ATP	adenosine triphosphate
BLCA	bladder urothelial carcinoma
BO-HEPES-IVM	HEPES-buffered in vitro maturation medium
BO-IVC	bovine embryo culture medium
BO-IVF	bovine in vitro fertilization medium
BO-SEMPREP	bovine semen preparation medium
BRCA	breast invasive carcinoma
C	control group
cDNA	complementary DNA
CCNB1	cyclin B1
cm	centimeter
COAD	colon adenocarcinoma
COCs	cumulus–oocyte complexes
CO2	carbon dioxide
DNA	deoxyribonucleic acid
DNMT3A	DNA methyltransferase 3 alpha
DPBS	Dulbecco’s phosphate-buffered saline
EEF1A1	eukaryotic translation elongation factor 1 alpha 1
FBS	fetal bovine serum
GLM	generalized linear model
GPX1	glutathione peroxidase 1
H19	H19 imprinted maternally expressed transcript
hpi	hours post-insemination
HSP	heat shock protein
HSP70	heat shock protein 70
HSP90	heat shock protein 90
HSP90AA1	heat shock protein 90 alpha family class A member 1
HSPB11	heat shock protein family B (small) member 11
HSPA1A	heat shock protein family A (Hsp70) member 1A
HS	heat-stressed group
HT	heat-treated group
ICM	inner cell mass
IGF2R	insulin-like growth factor 2 receptor
IETS	International Embryo Technology Society
IVEP	in vitro embryo production
IVF	in vitro fertilization
IVM	in vitro maturation
IVC	in vitro culture
LUAD	lung adenocarcinoma
mL	milliliter
miRNA	microRNA
mm	millimeter
mRNA	messenger RNA
ng	nanogram
O2	oxygen
OPU	ovum pick-up
PBS	phosphate-buffered saline
PEG	polyethylene glycol
PI3K	phosphoinositide 3-kinase
PLAC8	placenta-specific 8
qPCR	quantitative polymerase chain reaction
RNA	ribonucleic acid
ROS	reactive oxygen species
RPS15A	ribosomal protein S15A
RT	reverse transcription
SOM	self-organizing map
T/A	trading as
TE	trophectoderm
UTR	untranslated region
UV-Vis	ultraviolet–visible spectroscopy
